# Potential Antioxidant and Antiviral Activities of Hydroethanolic Extracts of Selected Lamiaceae Species

**DOI:** 10.3390/foods11131862

**Published:** 2022-06-24

**Authors:** Carmen Duque-Soto, Isabel Borrás-Linares, Rosa Quirantes-Piné, Irene Falcó, Gloria Sánchez, Antonio Segura-Carretero, Jesús Lozano-Sánchez

**Affiliations:** 1 Department of Food Science and Nutrition, University of Granada, Campus Universitario s/n, 18071 Granada, Spain; carmenduque@ugr.es (C.D.-S.); jesusls@ugr.es (J.L.-S.); 2 Department of Analytical Chemistry, Faculty of Sciences, University of Granada, 18071 Granada, Spain; ansegura@ugr.es; 3 Research and Development Functional Food Centre (CIDAF), Health Science Technological Park, Avenida del Conocimiento 37, Edificio BioRegión, 18016 Granada, Spain; rquirantes@cidaf.es; 4 Department of Preservation and Food Safety Technologies, Institute of Agrochemistry and Food Technology, IATA-CSIC, Av. Agustín Escardino 7, 46980 Paterna, Spain; irene.falco@iata.csic.es (I.F.); gloriasanchez@iata.csic.es (G.S.)

**Keywords:** phenolic compounds, polyphenols, medicinal and aromatic plants, HPLC-MS, antioxidant activity, antiviral activity, Lamiaceae

## Abstract

Medicinal and aromatic plants (MAPs) are potential sources of natural bioactive phytochemical compounds of an incredible worth for the food industry, such as polyphenols. Lamiaceae medicinal and aromatic plants from Granada’s high plateau, concretely *Origanum bastetanum*, *Thymus zygis gracilis*, *Thymus longiflorus*, *Thymus membranaceus* and *Ziziphora hispanica*, were evaluated under different conventional solid–liquid extraction conditions to obtain extracts enriched in bioactive compounds. Phenolic profile was detected by HPLC-QTOF-MS, identifying a high abundance of bioactive constituents. Furthermore, antioxidant and antiviral activities of the mentioned plants were studied as biological properties of interest for the improvement of food shelf-life. Thus, *Origanum bastetanum* showed the highest antioxidant potential for all assays. Antiviral activity was also tested against some important foodborne viruses, feline calicivirus (FCV), murine norovirus (MNV) and hepatitis A virus (HAV), with the highest activity obtained for *Ziziphora hispanica*, *Thymus longiflorus* and *Origanum bastetanum*. This research proposes the studied plants as rich sources of bioactive compounds with potential use as preservatives in the food industry.

## 1. Introduction

Since ancient times, plants such as herbs and spices have been used for a multitude of purposes, including nutrition and food preservation, either in their fresh crude form or as different preparations [1]. Thus, the historical use of medicinal and aromatic plants (MAP) has been widely acknowledged and recorded, as well as related to their chemical composition. These plants have proven to be an invaluable source of bioactive compounds with an incredible worth in modern industry. Herbs and spices are mainly differentiated based on the part of the plant from which they are obtained, being herbs mainly acquired from the leaves of a plant. Spices have traditionally been classified based on flavor into four groups: hot spices (such as black and white peppers), mild flavor spices (such as paprika), aromatic spices (such as clove or cinnamon) and aromatic herbs and vegetables (such as thyme) [2]. Among these, some substances have been used as fragrances and essences, industrial raw materials such as fatty acids and natural gums or even pesticides. In the food industry, these compounds have exerted an important role due to their preservative effect caused by the presence of antioxidant and antimicrobial constituents, which are considered some of the most interesting properties present in these plants.

Due to the interest in antioxidants for food applications and the recent search for novel and more natural sources of additives, in the last years, plants have been extensively studied for their antioxidant activity. MAPs constitute an attractive source not only for their availability and economical relevance, but also for the popularity of natural-based products in comparison with synthetically produced ones [3].

One of the most relevant antioxidant molecules found in MAP is polyphenols. These plant secondary metabolites are generally involved in defense against radiation, aggression by pathogens, or stress conditions, as they present a great antioxidant capacity [4,5]. In relation to this bioactivity, polyphenols have been proposed as alternatives to synthetic antioxidant compounds used in the food industry, such as butylated hydroxyanisole (BHA) or butylated hydroxytoluene (BHT). The trend for the use of alternative natural antioxidant is supported by the raising concerns about their harmful effects [3].

In addition to their antioxidant activity, some MAP extracts have shown activity against foodborne pathogens, such as viruses and bacteria, related to their phenolic content [6]. Therefore, plant-based extracts could also be introduced in processed foods in order to protect the consumers against foodborne viruses. According to the World Health Organization (WHO), almost one in ten people fall ill after consuming contaminated food across the world [7]. Consequently, its control during the production process has been a raising concern over the past years in the food industry. In fact, MAPs extracts are being studied as antiviral agents against a wide variety of viruses responsible for foodborne illnesses [8].

Being one of the most important herbal families, the Lamiaceae family, including oregano, rosemary and thyme, pose as an interesting source of extracts owing to their naturally high phenolic content [9]. These species possess multiple activities from antioxidant and anti-inflammatory to antibacterial and antiviral properties [1]. In this sense, *Thymus*, *Origanum* and *Ziziphora* genus stand out due to their extensive use for culinary purposes, being popular additions in food preparations. As reported in previous literature, other spices such as clove and cinnamon have shown great antioxidant activity, which has been even used for medicinal purposes [10,11,12]. Additionally, clove and ginger have also presented antiviral activity against food-borne viruses such as feline calicivirus [13]. Therefore, phenolic extracts of these spices could exhibit bioactive properties of great importance for their use the food industry. However, current compositional data and studies regarding these properties are still insufficient.

The purpose of this study was to establish the phenolic profile, antioxidant capacity and antiviral activity against foodborne norovirus surrogates (feline calicivirus, FCV; murine norovirus, MNV; and hepatitis A virus, HAV) of optimized phenolic-rich extracts of a variety of MAPs from the Lamiaceae family (*Origanum bastetanum*, *Thymus zygis gracilis*, *Thymus membranaceus*, *Thymus longiflorus* and *Ziziphora hispanica*), typical from Granada’s high plateau. This study aimed to find new potential sources of natural bioactive compounds in this Mediterranean region as well as to evaluate their mentioned bioactivity in relation to their phenolic content.

## 2. Materials and Methods

### 2.1. Chemicals and Reagents

All reagents and solvents were of analytical or MS grade. For extraction, ultrapure water was obtained with a Milli-Q system (Millipore, Bedford, MA, USA) and absolute ethanol (EtOH) was purchased from Fisher Scientific (Loughborough, Leicestershire, UK). Regarding HPLC-MS analysis, LC–MS grade acetonitrile was purchased from Fisher Scientific (Loughborough, Leicesterchire, UK), formic acid was supplied by Sigma-Aldrich (Buchs, Switzerland) and ultrapure water was obtained as described above. In order to measure the antioxidant capacity of the MAP extracts, the following reagents were provided by the indicated suppliers: AAPH (2,20-azobis-2-methyl-propanimidamide, dihydrochloride), ABTS [2,20-azinobis (3-ethylbenzothiazoline-6-sulphonate)], fluorescein, potassium persulfate, TPTZ (2,4,6-tripyridyl-S-triazine) and Trolox (6-hydroxy-2,5,7,8-tetramethylchroman-2-carboxylic acid) from Sigma-Aldrich (St. Louis, MO, USA). From Panreac (Barcelona, Spain), sodium phosphate mono and dibasic, sodium acetate, ferric chloride, hydrochloric acid and acetic acid were purchased. For antiviral activity, Dulbecco’s Modified Eagle’s Medium and fetal bovine serum were provided by Labclinics (Biowest, L0101-500) and Fisher (Invitrogen, 10309433), respectively. In these assays, a commercial rosemary extract already used as food additive obtained from a commercial supplier (NATAC Group S.L., Madrid, Spain) was used as reference sample with accredited food additive properties.

### 2.2. Samples

The samples of *Origanum bastetanum*, *Thymus zigis gracilis*, *Thymus membranaceus*, *Thymus longiflorus* and *Ziziphora hispanica* were collected during April and May of 2020 in different sites in the province of Granada (Spain) belonging to the area of Granada’s High Plateau. Samples of all the studied plants were authenticated by a botanist and a voucher specimen of each one was deposited in the herbarium of the University of Granada located in Rector López Argüeta street, number 8, PC: 18,001 (Granada). The assigned codes in the herbarium were 69,287 for *Origanum bastetanum,* 69,289 for *Thymus zigis gracilis*, 69,290 for *Thymus membranaceus*, 69,288 for *Thymus longiflorus* and 69,286 for *Ziziphora hispanica*. The information regarding altitude, temperatures, rain water and solar radiation of this area is depicted in Table S1, in Supplementary Materials. After their collection, the samples were dried following the traditional methodology of maintaining them at room temperature and darkness for 30 days. After the drying step, the samples were grounded with an ultra-centrifugal mill ZM 200 (Retsch GmbH, Haan, Germany) obtaining powders with an average particle size of 500 µm. The obtained material was stored, avoiding light and air exposure, and kept at room temperature until their extraction.

### 2.3. Conventional Solid–Liquid Extraction

Sample extraction was carried out by maceration with solvents considered as Green (environmentally friendly) and GRAS (Generally Recognized As Safe), allowed for food use. In this sense, different proportions of two solvents with these characteristics, water and ethanol, were assayed. Concretely, pure solvents and aqueous mixtures with 50 and 80% ethanol were tested. For all the experiments, 50 mL of the extraction solvent was mixed with 1.5 g of each dry plant, and the mixture was shaken in a vortex for 30 s. After that, solutions were maintained in agitation at room temperature for 1 h. Supernatants were removed with two subsequent centrifugation cycles at 12,096× *g* RCF for 10 min. Liquid extracts were evaporated to dryness at 35 °C in a Savan SC250EXP Speed-Vac (Thermo Scientific, Loughborough, Leicestershire, UK). Extracts were stored at −20 °C until further use. All the extractions were performed in duplicate in order to assure repeatability. Extraction yields are presented in Table S2, in Supplementary Materials.

### 2.4. Analysis by HPLC-QTOF-MS

Extracts were analyzed at a concentration of 5 g/L prepared in the same solvents used for their extraction. Solutions were filtered using syringe filters of 0.2 µm pore size. Samples were analyzed in a 1260 HPLC instrument coupled to a 6540 UHD Quadrupole-Time-Of-Flight mass analyzer (QTOF-MS) from Agilent Technologies (Palo Alto, CA, USA).

The chromatographic method was optimized for the separation of the analytes in 10 µL of sample with a 1.8 µm Zorbax Eclipse Plus C18 column (150 × 4.6 mm). The mobile phases for the elution were 0.1% aqueous formic acid as phase A and acetonitrile as phase B. The gradient elution started at 5% B, the first step reached 60% B at minute 30, followed by 95% B after 35 min. After that time, the initial conditions were restored in 5 min and maintained for another additional 5 min before the next injection to equilibrate the system. The temperature of the oven was maintained at 25 °C during the analysis while the flow was set at 0.5 mL/min.

For the detection in the mass spectrometer, both the ionization and the transfer parameters were optimized. The instrument was equipped with a Jet Stream dual ESI interface and nitrogen was used as drying and nebulizing gas. The flows and temperatures of nebulizer and drying gas were: 20 psig nebulizer, 10 L/min drying gas at 325 °C and 12 L/min of sheath gas at 400 °C. The applied voltages were: capillary 4000 V, nozzle 500 V, fragmentor 130 V, skimmer 45 V, octopole 1 RF 750 V. Regarding the acquisition parameters, the registered *m*/*z* range was 100–1700 *m*/*z*, the acquisition rate and time were 3 spectra/min and 333.3 ms/spectra, respectively. The acquisition mode was negative ionization with the continuous infusion of the reference ions *m*/*z* 112.985587 (trifluoroacetate anion) and 1033.988109 (adduct of hexakis (1H,1H,3H-tetrafluoropropoxy) phosphazine or HP-921) to correct each mass spectrum in order to achieve high accurate mass measurements.

All the operations, acquisition and data analysis were controlled by Masshunter workstation software version B.06.00 (Agilent Technologies, Palo Alto, CA, USA). This software package uses The FindCompounds algorithms, which find compounds in data and creates averaged MS spectra for each compound. This functionality is an easy way to “mine” information from complex data. In this research the analysts used the “Find Compounds by Molecular Feature” tool and extracted the complete result set for a compound. After that, the software generated possible formulas for each of those found compounds, together with other information, such as Score (%) or Error mass (ppm and mDa), which result in potential candidates that should be checked in a database. In this step, the analysts also checked the predicted isotope abundance ratios on the spectrum plot. Lastly, all those candidates were searched in the SciFinder database, filtering by the spice of the plant family of interest. The positive results were included as tentative identified compounds. The area under the peak of each compound tentatively identified was measured in the base peak chromatogram of each plant matrix. This area was obtained for each analysis replicate for all the studied plants, and summarized in Table S3 as mean value ± standard deviation.

### 2.5. Evaluation of Potential Bioactivity

Different analyses were performed in order to evaluate the antioxidant and antiviral activities of the obtained MAP extracts for exploring possible food and pharmaceutical applications. In this sense, different antioxidant activity assays (FRAP, TEAC, ORAC) were carried out in order to evaluate different antioxidant mechanisms from bioactive substances (electron transfer, hydrogen transfer or the combination of both). Moreover, the antiviral activity of these Lamiaceae plants was tested in three different virus cell lines.

#### 2.5.1. In Vitro Antioxidant Activity

A variety of antioxidant assays (TEAC, FRAP, and ORAC) were evaluated for the studied herbs extracted with different hydroethanolic mixtures (ethanol; 80% ethanol; 50% ethanol and water). Additionally, a commercial rosemary extract (reconstituted in ethanol and aqueous ethanol at 80%) was used as positive control.

The TEAC (Trolox Equivalent Antioxidant Capacity) assay was performed as previously described elsewhere [14] with slight modifications [15]. This method assesses the ABTS radical cation (ABTS+•) scavenging activity of samples compared to a hydrophilic analogous of vitamin E, Trolox. Briefly, the ABTS+• stock solution was prepared by mixing 7 mM aqueous ABTS solution with 2.45 mM potassium persulfate. After 12–24 h in darkness at room temperature, the ABTS+• solution was diluted with H_2_O:EtOH (1:1, *v*/*v*) to adjust its absorbance value to 0.70 ± 0.02 at 734 nm. A volume of 20 µL of diluted samples was then mixed with 200 µL ABTS+• working solution in a 96-well microplate and the decay in absorbance after 30 min at 25 °C was monitored in a microplate reader from Synergy MX, BioTek (Winooski, VT, USA). A standard curve with Trolox was prepared to express the antioxidant activities as µmol Trolox equivalents/mg dry extract (DE).

The FRAP (Ferric Reducing Antioxidant Capacity) assay was carried out following the method described by Benzie and Strain (1996) [16] with slight modifications. This method is based on the ability to reduce the ferric to ferrous cation in acidic medium by antioxidant substances. In this sense, 40 µL of diluted samples was put into 250 µL of a mixture of 300 mM sodium acetate (pH 3.6 with acetic acid), 10 mM TPTZ (40 mM aqueous hydrochloric acid) and 20 mM aqueous ferric chloride. FRAP values (expressed as µmol FeSO_4_ equivalents/mg dry extract) were calculated by measuring the absorbance before and after the addition of the sample of the ferrous complex at 593 nm in a microplate reader from Synergy MX, BioTek (Winooski, VT, USA) and using FeSO_4_·7H_2_O as standard.

To assay the capacity of the extracts to scavenge peroxyl radicals, an ORAC (Oxygen Radical Absorbance Capacity) method was used [17] with some modifications [18]. This assay measures the decrease in fluorescence of a protein as a result of the loss of its conformation when it undergoes oxidative damage caused by a source of peroxyl radicals (ROO•). The ORAC in vitro assay tests the ability of a sample to inhibit the reactivity of these free radicals to be quantified. Specifically, it measures the capacity to capture a specific radical, peroxyl, generated from the organic molecule AAPH. The measurements were made in a microplate reader Synergy MX, BioTek (Winooski, VT, USA) with an excitation and emission wavelengths of 485 and 520 nm, respectively. A regression equation between the Trolox concentration and the net area of the fluorescence decay curve was used in order to obtain the final ORAC values, expressed as µmol Trolox equivalents/mg dry extract. In all the antioxidant capacity assays, measurements were made in triplicate and the final value is expressed as the media ± standard deviation of the three replicates.

#### 2.5.2. Antiviral Activity

Three different viruses and their respective cell lines were used to evaluate the potential antiviral activity of the MAP extracts with the highest antioxidant. Thus, the extracts obtained with 80% ethanol were chosen for *Origanum bastetanum*, *Thymus zigis gracilis* and *Thymus membranaceus*, whereas the aqueous mixtures at 50% were selected for *Thymus longiflorus* and *Ziziphora hispanica*, together with the ethanolic extract of *Thymus zigis gracilis*. FCV F9 strain (ATCC VR-782), MNV-1 (kindly provided by Prof. H.W. Virgin, Washington University School of Medicine, USA) and HAV strain HM-175/18f (ATCC VR-1402) were assayed and propagated in CRFK (ATCC CCL-94), RAW 264.7 (also provided by Prof. H.W. Virgin) and FRhK-4 cells (provided by Prof. A. Bosch, University of Barcelona, Spain), respectively. To produce virus stock, cell lines were infected for 2 days with FCV and MNV and for 15 days with HAV followed by three thaw cycles at 660× *g* for 30 min. Infectious viruses were enumerated by determining the 50% tissue culture infectious dose (TCID50) using the Spearmen–Karber method [19].

Suspensions of FCV, MNV and HAV were equally mixed with extracts of *Thymus zygis gracilis*, commercial rosemary sample (previously suspended in ethanol or 80% aqueous mixture), *Ziziphora hispanica*, *Thymus longiflorus* (50% ethanol), *Origanum bastetanum* and *Thymus membranaceus* (80% ethanol) extracts at two different concentrations (0.5 or 5 mg/mL). Samples were incubated overnight at 25 °C for FCV or 37 °C in the cases of MNV and HAV, and then Dulbecco’s Modified Eagle’s Medium supplemented with 10% fetal bovine serum was added to stop the reactions. Each treatment was run in triplicate. Positive controls were virus suspensions mixed with PBS only under the same experimental conditions. Samples were diluted and inoculated into confluent cell lines and infectious viruses were enumerated as described above. The decay of virus titers was calculated as previously described [20].

All antiviral samples were compiled from three independent experiments with three technical replicates for each variable. To test the impact of each variable on viral infectivity results, data were subjected to analysis of variance (ANOVA) followed by Tukey’s HSD as a post hoc test to obtain homogenous groups. Differences in means were considered significant when the *p*-value was < 0.05.

## 3. Results

### 3.1. Characterization of Bioactive MAP Extracts by HPLC-QTOF-MS

**Figure 1:**
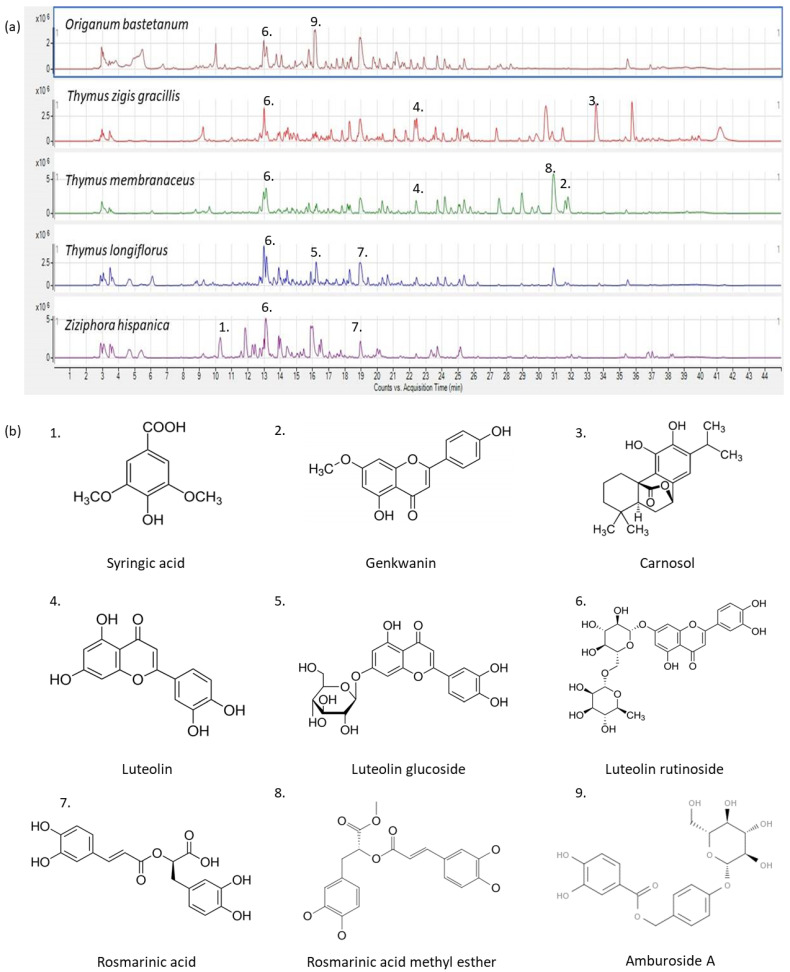
(**a**) Base Peak Chromatograms of MAP extracts with higher bioactivity obtained by HPLC-QTOF-MS; (**b**) chemical structure of some compounds identified in the selected extracts.

Once bioactivity assay screening was performed with the obtained extracts, those that exerted the highest potential were comprehensively characterized by HPLC-QTOF-MS. Thus, the analyzed extracts were *Origanum bastetanum*, *Thymus zygis gracillis* and *Thymus membranaceus* extracted with 80% aqueous ethanol, and *Thymus longiflorus* and *Ziziphora hispanica* (obtained with 50% ethanol-water). The obtained base peak chromatograms (BPC) using the powerful analytical platform are depicted in Figure 1.

As it can be observed, the chromatographic profiles were complex, with the thyme species being very similar except for the less polar substances eluting at the end of the chromatographic run. The tentative identification of the detected compounds is summarized in Table 1, where the putative identity of the tentative compounds, their retention time, experimental m/z, molecular formula, score and error (ppm) are presented together with the information regarding their presence in the different analyzed MAP extracts in which they were found. Thus, *Origanum bastetanum* is referred to as OG, *Thymus zygis gracillis* as TG, *Thymus membranaceus* as TM, *Thymus longiflorus* as TL and *Ziziphora hispanica* as Z; while common compounds were expressed as “All” in the “MAP extract” column. A high abundance of bioactive compounds was found for all extracts, mainly belonging to phenolic acid and flavonoid families.

**Phenolic acids and derivatives**. Many of these compounds were previously found in different species of Lamiaceae, such as protocatechuic acid hexoside, syringic acid, chlorogenic acid, rosmarinic acid and its methyl ester derivative [21,22]. Additionally, coumaroylquinic acid isomers and feruloylquinic acid have been previously identified in Lamiaceae plants [23]. Rosmarinic and chlorogenic acid were also described for different species of *Ziziphora* [23].

**Lignans**. Several salvianolic acids and their isomers were also identified, described and studied in previous studies of *Thymus* and *Origanum* [24]. Salvianolic acid compounds have demonstrated bioactivity such as antioxidant activity [25].

**Flavonoids**. Some of these compounds included different isomers of luteolin rutinoside, luteolin glucoside and luteolin, all of them previously described for other species of thyme and oregano [26]. Apigenin glucuronide, apigenin and genkwanin were also identified, all of them described in other species of thyme by other authors [21,22,27]. Other flavones proposed were cirsimaritin isomers, cirsiliol, thymusin and hispidulin, substances previously found in thyme [27,28].

Furthermore, the flavanones eriodictyol and its glucoside form [28], naringenin [27] and dihydroflavonol taxifolin [22] have been described in previous research in thyme. The flavan-3-ols gallocatechin and epigallocatechin have also been identified in Lamiaceae in the literature [28].

Other phenolic compounds have also been described in diverse Lamiaceae plants, such as phenolic glycosides like leonuriside A, seguinoside K [29] and amburoside A [30], phenyl propanoids such as caffeoylquinic acid methyl ester and dicaffeoyl-hydroxy-methylglutaroyl-quinic acid, simple phenols like piceol, mono terpenes (thymohydroquinone acetylglucoside), and phenolic diterpenes such as carnosol [21].

The number of the compounds tentatively identified in the MAP extracts should be highlighted. Thus, in *Origanum bastetanum*, a total of 20 compounds were proposed, 22 substances in *Ziziphora hispanica*, 28 in *Thymus zigis gracilis*, 34 in *Thymus membranaceus*, and lastly 39 in *Thymus longiflorus*.

A great number of compounds were only detected in *Origanum bastetanum*, which makes this species one of most interesting among the studied MAPs. Some of these compounds include salvianolic acid A and B and other derivatives of caffeic and quinic acids, among others. Moreover, this extract also presented substances in common with thyme species, such as cirsimaritin isomers, trihydroxyoctadecadienoic acid, eriodictyol isomer or the glucosilated form of tuberonic acid (also found in *Ziziphora hispanica*). Most of these compounds have been previously identified in species of oregano or in the Lamiaceae family, as previously mentioned. On the contrary, as far as we are concerned, caffeic acid derivatives and syringic acid glucoside were detected for the first time in the present research.

On the other hand, *Ziziphora hispanica* also possesses an abundance of phenolic compounds. Specifically, 11 molecules have been exclusively detected in this plant, such as caffeic and quinic acid derivatives, along with other compounds such as barosmin or botcinin acid. Furthermore, some of the putative compounds in *Ziziphora hispanica* were also found in *Thymus longiflorus*, which could be explained by the similarity in their composition as well as the use of the same solvent conditions for their extractions (water-ethanol, 50:50, *v*/*v*). Those common compounds were principally polar substances, such as citric acid isomers, xantone, rosmarinic acid and gallocatechin. Furthermore, other tentative identified compounds were also characterized for thyme species, specifically tuberonic acid glucoside, syringic acid and luteolin. Due to the scarce bibliographic information of this aromatic plant, just the isomers of citric and chlorogenic acids, piceol, basrosmin, rosmarinic acid and luteolin have been reported in this species [23], whereas the rest belonged to the Lamiaceae family except for p-coumaroylquinic acid and botcinic acid, which are frequently found in other plants.

In relation to the thyme species, seven compounds were found in all the analyzed thyme samples. Other substances were only found in two out of the three samples of thyme. On the one hand, carnosol and one isomer of tuberonic acid glucoside were detected in *Thymus zigis gracillis* and *Thymus longiflorus*; whereas epigallocatechin, taxifolin, thymusin, tuberonic acid and rosmarinic acid methyl ester were identified in the first species and *Thymis membranaceus*. On the other hand, *Thymus membranaceus* and *Thymus longiflorus* possessed mainly flavonoids in common. Finally, as can be seen in Table 1, different compounds were exclusively proposed for some of the analyzed thyme samples, concretely 5 for *Thymus zigis gracillis*, 4 in *Thymus longiflorus* and 2 in *Thymus membranaceus*. The scarcity of literature regarding the characterization of the considered spices should be noted, while also considering their antioxidant and antiviral activities, showing the novelty of the present study.

### 3.2. In Vitro Antioxidant Activity

The results of the different antioxidant assays performed in the aromatic plant extracts are summarized in Table 2. The values of the measurements are expressed as µmol Trolox equivalents/mg dry extract for TEAC and ORAC assays, and as µmol FeSO_4_ equivalents/mg dry extract for the FRAP test.

Regarding the TEAC assay, it should be noticed that ethanol–water at a proportion of 80:20 (*v*/*v*) seems to be the best solvent to extract antioxidant substances from all the aromatic plants. Moreover, *Origanum bastetanum* presents the highest antioxidant activity for this assay, followed by *Thymus zygis gracilis* and *Thymus longiflorus*. Additionally, *Ziziphora hispanica* possessed the least antioxidant activity against ABTS+• radicals.

Concerning FRAP measurements, similar antioxidant extraction efficiency with the assayed solvents was observed. The extracts obtained with 80% ethanol presented the best antioxidant results for *Origanum bastetanum* and the species *Thymus zygis gracilis* and *Thymus membranaceus*; whereas a mixture of water–ethanol at 50% extracted more quantity of antioxidants of *Thymus longiflorus* and *Ziziphora hispanica*. In this assay, the best antioxidant capacity was again shown by *Origanum bastetanum*, followed by *Thymus longiflorus* extracted with the hydroethanolic mixtures.

As for the ORAC test, the assayed hydroalcoholic solvents were the best option for the recovery of antioxidant compounds, except for *Thymus zygis gracilis*, for which pure ethanol showed the best results. Higher percentages of ethanol in aqueous mixtures appeared to be better for *Origanum basetanum* and *Thymus membranaceus*, while the 50% proportion seemed to be optimum for *Thymus longiflorus* and *Ziziphora hispanica*. In the same way, the highest antioxidant potentials were exhibited by *Origanum bastetanum* and *Thymus zygis gracilis*.

In general, it should be pointed out that the aqueous mixtures with ethanol present the best results with respect to the antioxidant activity of the assayed MAP extracts. Furthermore, *Origanum bastetanum* had the highest antioxidant potential in all the assays, followed by the different species of thymes, mainly *Thymus longiflorus* and *Thymus zygis gracilis*.

### 3.3. Antiviral Activity

The aim of this study was to evaluate the antiviral activity of selected MAP extracts against different enteric viruses and their cultivable surrogates, as a form of evaluating their use for protecting consumers against some of the most important foodborne viruses. In this sense, extracts presenting the best results in terms of antioxidant activity were selected for this evaluation. Thus, extracts obtained with 80% ethanol were chosen for *Origanum bastetanum*, *Thymus zigis gracilis* and *Thymus membranaceus*, whereas aqueous mixtures at 50% were selected for *Thymus longiflorus* and *Ziziphora hispanica*, together with the ethanolic extract of *Thymus zigis gracilis*. The studied MAPs were compared with a commercial rosemary extract (reconstituted in ethanol and aqueous ethanol at 80%), used as positive control due to its proven activity being previously evaluated by the EFSA Panel on Food Additives, Flavorings, Processing Aids and Materials in contact with Food [31] and its authorization for use as a dietary antioxidant additive in the European Union (EU) according to Annexes II and III to Regulation (EC) No 1333/200822 [32].

**Figure 2:**
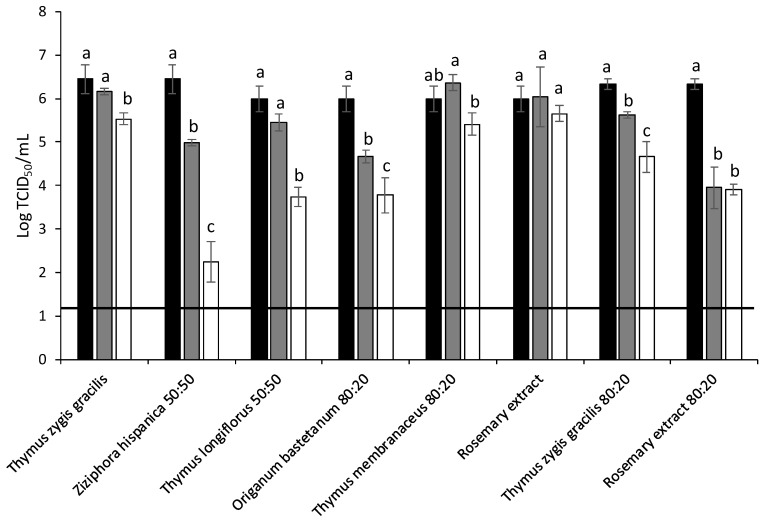
Reduction of feline calicivirus (FCV) titers (log TCID50/mL) treated with different extracts after 25 °C/ON incubations. Black bars: Control; Grey bars: 0.5 mg/mL; White bars: 5 mg/mL. Each bar represents the average of triplicates. Within each column, different letters denote significant differences between treatments. Horizontal line depicts the detection limit.

**Figure 3:**
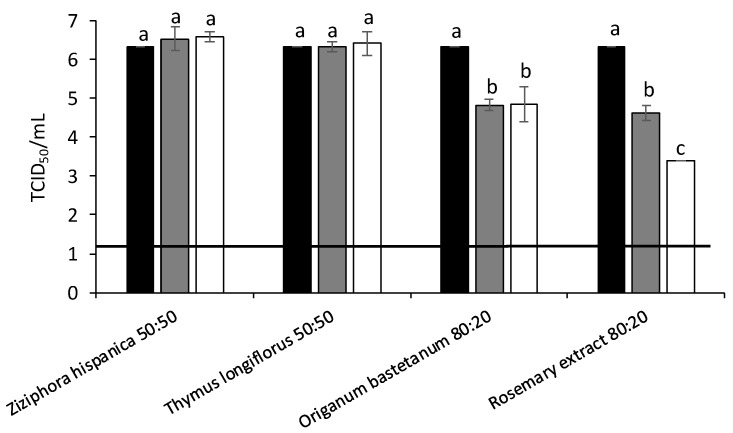
Reduction of murine norovirus (MNV) titers (log TCID50/mL) treated with different extracts after 37 °C/ON incubations. Black bars: Control; Grey bars: 0.5 mg/mL; White bars: 5 mg/mL. Each bar represents the average of triplicates. Within each column, different letters denote significant differences between treatments. Horizontal line depicts the detection limit.

**Figure 4:**
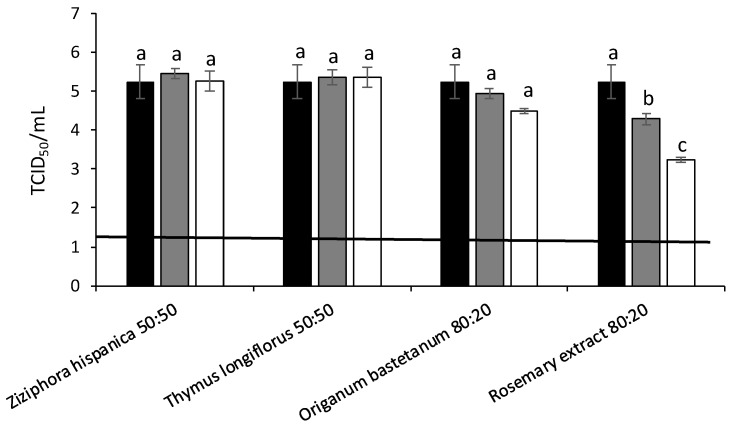
Reduction of hepatitis A virus (HAV) titers (log TCID50/mL) treated with different extracts after 37 °C/ON incubations. Black bars: Control; Grey bars: 0.5 mg/mL; White bars: 5 mg/mL. Each bar represents the average of triplicates. Within each column, different letters denote significant differences between treatments. Horizontal line depicts the detection limit.

Initially, FCV were treated at 25 °C with the different MAP extracts. The highest antiviral activity was obtained for *Ziziphora hispanica*, *Thymus longiflorus*, *Origanum bastetanum* and commercial rosemary (80%) extracts reducing FCV titers by 4.21, 2.25, 2.21 and 2.42 log at 5 mg/mL, respectively (Figure 2). The antiviral activity of these four extracts was further evaluated against MNV (Figure 3) and HAV (Figure 4) at 37 °C. In the case of MNV, significant differences were observed for *Origanum bastetanum*, getting reductions by 1.50 and 1.48 log at 0.5 and 5 mg/mL, respectively, and 1.71 and 2.92 log for rosemary extract at the same concentrations. While for HAV, only the rosemary extract showed antiviral activity, reducing titers by 0.96 and 2.92 log for lowest and highest concentrations, respectively.

## 4. Discussion

Medicinal and aromatic plants’ potential as sources of natural bioactive compounds makes their study of high interest. In this research, phenolic content and bioactivity of some of the most remarkable MAPs found in Granada’s high Plateau were evaluated. It should be highlighted that the identification of the phytochemicals present in the studied plants is only tentative, and a confirmation should be performed by the comparison with commercial standards when available for the compounds of interest.

Bioactive composition of the studied plants has shown a great variety of phenolic compounds, as can be observed in the previous section. The uniqueness of their polyphenolic profile must be considered, as *Origanum bastetanum* extract presented an abundance of compounds only identified in this matrix, while 11 compounds were exclusive for *Ziziphora hispanica* and similarity was found between thyme species. This variety generates individual combinations of phenolic compounds for each plant, which have proven to be of high interest.

Different species of *Origanum* have been studied in the literature, mainly focusing on *Origanum vulgare.* As for *Origanum bastetanum*, its essential oil has been previously studied but, to our current knowledge, aqueous extracts have not been described in literature. The phenolic profile of the studied extract seems to deviate from that observed in the literature for *Origanum vulgare*, *O. dictamnus* and *O. majorana.* The phenolic profiles studied for *O. vulgare* by Zhang et al., 2014, Vallverdú-Queralt et al., 2014 and Shan et al., 2005, differ from that observed in our study, with only derivatives from caffeic acid and amburoside A being in common [13,21,30]. This can be extended to *O. dictamnus* and *O. majorana*, sharing only eriodictyol, syringic acid glucoside, caffeoyl arbutin and salvianolic acid [22,33]. However, some of the compounds described in the literature for oregano have been observed in other species identified in this study, such as apigenin (TG), carnosol (TG and TL) and chlorogenic acid (Z). These differences could be related to the fact that those studies considered other species of *Origanum*. Nevertheless, the influence of the pedoclimatic conditions characteristic from Granada’s High Plateau, which combines altitude and a high exposure to UV radiation, could also have an influence on its specific phenolic composition. Thus, differences in genus and cultivar may be responsible for these variations, as the *Origanum* spices found in the literature come from different varieties and geographical points.

On the other hand, while maintaining differences in phenolic composition, thyme seems to have more compounds in common with other studied species. In Nabet et al., 2019, *Thymus fontanesii* was studied, which presented caffeic acid, gallocatechin (TL), rosmarinic acid, other forms of luteolin, apigenin, naringenin and carnosol. Other studies have considered *Thymus vulgaris*, sharing some compounds with the present extracts, mainly phenolic acids such as caffeic and rosmarinic acid, as well as naringenin, apigenin and luteolin derivatives [13,21,34,35]. Nonetheless, most of the compounds identified in the present study have not been recorded for *Thymus vulgaris*, such as genkwanin and most of the caffeic derivatives identified. As previously mentioned, some of these compounds have already been described for other Lamiaceae plants, but to our knowledge, they are rare in *Thymus*. The geographical effect could also be an explanation for the observed differences, as those found in the literature come from different locations. This could be supported by Horwath et al., 2008, where phenolic compounds like genkwanin were observed in wild populations of *Thymus* from climatic regions in the southeast of Spain.

This fact has also been observed for *Ziziphora*, where only some compounds have been described in the literature, such as chlorogenic acid, luteolin and syringic acid [12].

Bioactivity of the studied extract has shown to be quite promising. Overall, antioxidant activity of the obtained results seems to be high and consistent with previous published research for some species of Lamiaceae [36,37,38]. Moreover, this study presents promising results of antioxidant potential for these spices. In this sense, the present antioxidant results appear to be high when compared with acclaimed high antioxidant sources such as rosemary extracts, with a range of 3.1 to 7.4 mmol TE/kg FW for TEAC [39] and ranging from 32.17 to 1.28 μm Fe (II)/g for FRAP [40]. The obtained data is also higher than those obtained in Nabet et al., 2019, where other types of oregano and thyme reported values of 3.61 mmol Trolox equivalents/g and 3.92 mmol Trolox equivalents/g for TEAC, respectively. In Vallverdú-Queralt et al., 2014, oregano and thyme were also studied, obtaining results of 1.34 ± 0.13 and 1.38 ± 0.13 mmol TE/g DW for ABTS, as well as 0.78 ± 0.07 and 1.15 ± 0.06 mmol TE/g DW for DPPH, respectively. In Miron et al., 2011, antioxidant capacity of wild thyme was studied for different extraction conditions, with the highest result being 3.08 ± 0.09 mmol Trolox/g when using water/ethanol 50:50 (*v*/*v*) as solvent. The presented results show an incredible potential of the mentioned phenolic extracts as additives for preservation of food quality and safety and improving shelf-life.

As rosemary phenolic extracts have already been approved as applicable additives, the fact that the studied Lamiaceae extracts seem to show comparable if not a higher antioxidant activity demonstrates the adequacy for their potential use in the food industry. This puts extracts such as *Origanum bastetanum,* which has shown the greatest antioxidant results for all the considered assays, at the level of recognized additives, implying its outstanding potential as a novel source of active compounds for the food industry. Additionally, its use will suppose a new area of exploitation for Granada’s agricultural businesses.

As for antiviral activity, the scarcity of studies carried out for MAPs phenolic aqueous extracts of the Lamiaceae family must be highlighted. Thus, the present results have also been compared with those of other plant sources. Through the literature, similar tendencies were previously observed in oregano essential oil (*Origanum vulgare*), which successfully deactivated feline calcivirus (FCV) and murine norovirus (MNV). This activity has been extended to the studied phenolic extract, as observed in the present research. These results are also comparable with previous research on other plant extracts. In Li et al. (2012), a reduction to >3-log PFU/mL of MNV was observed when grape seed extract was tested [41]. Similarly, Su and D’Souza (2013) [26] observed a reduction of FCV-F9 by 2.71 log on lettuce and 3.05 log on peppers and a reduction of MNV by 0.2–0.3 log on lettuce. These findings are comparable with those from this study for FCV and lower in the case of MNV.

These results reflect the possible potential of the studied extracts. This is especially important for FCV, where a higher reduction is observed for *Ziziphora hispanica* in comparison with the commercial rosemary extract, while *Thymus longiflorus* and *Origanum bastetanum* also presented significant activity. Furthermore, in the case of MNV presence, the results for *Origanum bastetanum* extracts are relatively close to the observed for the assayed rosemary extract, which put into light the potential use of this oregano as additive. Therefore, the antiviral effects observed for the aforementioned extracts shows their ability to reduce the presence of FCV or MNV in food products, as well as its role in preventing the transmission vectors for those viruses. This fact is mainly achieved for *Origanum bastetanum*, whose extracts are successful in the reduction of both viruses.

As it could be observed, the Lamiaceae family presents outstanding potential as a source of bioactive compounds with great potential as preservative additives for the food industry. Nevertheless, the literature regarding the bioactive composition of these MAPs is mainly focused on the study of their essential oils, with the polar phenolic extracts being understudied. This fact hinders the accurate comparison and correlation of data with these polar extracts and endorses the importance of the present investigation and results.

Bioactivity of the considered extracts may be related to each identified bioactive compound profile through the structure of each of their present compounds. Thus, structure–activity relationships of the phenolic compounds identified was also considered for this study.

Antioxidant activity of phenolic acids has been observed in previous studies as directly related to the number and position of hydroxyl groups. Particularly, previous studies have shown that phenolic compounds presenting a second hydroxyl group in the ortho- or para- position seem to show higher antioxidant capacity that those in the meta- position [42]. This can be explained by the strong electron donating ability of these forms. The detachment of the H atom during antioxidant reactions leads to the formation of phenoxyl radicals, which can be stabilized through the inductive or resonance effect [43]. This phenomenon results in the low activation energy of the transfer of the second phenolic H atom, thus enhancing their antioxidant activity, as seen for catechol groups [44]. Therefore, the ability to donate a hydrogen as well as stabilization of the resulting phenolic radical by electron delocalization are essential to the development of high free radical scavenging activity.

This structure can be found in many of the proposed compounds, as for protocateuchuic, chlorogenic, rosmarinic or caffeic acids. Moreover, it was especially present in the extracts with the highest antioxidant activity, such as *Origanum bastetanum*. In addition, some of these substances have been previously reported for this plant and directly associated with a significant antioxidant activity [21]. Furthermore, the presence of compounds presenting three catechol groups in their structure should be mentioned in *Origanum bastetanum* extract, especially the identified salvianolic acids A and B isomers (with the highest abundance in *Origanum bastetanum*), dicaffeoyl-hydroxy-methylglutaroyl-quinic acid and luteolin rutinoside. Therefore, it can be assumed that these compounds may contribute to its greater activity.

Phenolic structures with other functional groups, such as OAc or C=O (oxo) have also been only found in *Origanum bastetanum* extract in high abundance, such as Leonuriside A or Seguinoside K, which may also be related to its high antioxidant activity. Nevertheless, it has been reported that their presence contributes to the free radical scavenging activity to a lesser extent [45].

Additionally, flavonoids are abundant in the studied samples. Their high free radical scavenging activity results from the location and presence of hydroxyl and oxo groups and double bonds [43]. Firstly, the ortho-dihydroxy group (catechol) in the B-ring, as previously discussed, ensures the high stability of phenoxyl radicals, thus enhancing its ability to scavenge free radicals, as found in luteolin, taxifolin or epigallocatechin (abundant in *Thymus zygis gracilis* and *membranaceus*), luteolin rutinoside (with a remarkable abundance in all the studied samples), eriodictyol (mainly present in *Thymus* varieties and *Origanum bastetanum*), cirsiliol (with higher presence in *Thymus longiflorus* and *membranaceus*), and gallocatechin (with a highlighting abundance in *Thymus zygis gracilis* and *Ziziphora hispanica*).

Secondly, the presence of an unsaturated bond between C2 and C3 in conjugation with a 4-oxo group in the C-ring is found in compounds such as luteolin, luteolin rutinoside, cirsimaritin (showing a higher presence in all the varieties of *Thymus* and *Origanum bastetanum*), apigenin (being more abundant in *Thymus longiflorus* and *membranaceus*), apigenin glucuronide (mainly found in *longiflorus*) and cirsiliol [43]. This structure may result in higher antioxidant properties due to the dislocation of an electron in the B-ring, stabilizing the resulting phenoxyl radical. Lastly, C-rings with 4-oxo groups and OH groups near C3 and C5 are able to generate high free radical scavenging activities [43]. This is the case for luteolin, luteolin rutinoside, eriodictyol, cirsimaritin, taxifolin, apigenin, apigenin glucuronide, cirsiliol, and naringenin.

The aforementioned flavonoids are abundant in *Thymus zygis gracilis* and *Thymus longiflorus* extracts, both showing significant antioxidant activity, but they are also present in *Origanum bastetanum* extract. Nevertheless, even if some of these compounds are also found in other extracts, we must take into consideration that the antioxidant activity of phenolic plant extracts is not only due to the effect of a single antioxidant agent, but to the interaction between its different compounds. It is safe to assume that the abundance, structural nature and synergistic effect of the unique combination of phenolic compounds in the oregano extract are important contributors to its greater antioxidant activity compared to the other MAP extracts analyzed in this study.

As for the relationship between structure and antiviral activity, it has been previously reported in the literature that the presence and position of hydroxyl and ester groups is necessary for antiviral activity, as for flavanones [46]. This could be the case for taxifolin, whose activity seems to be related to the presence of an OH group at the C3′ position [46]. The presence of six hydroxyl groups in epigallocatechin and gallocatechin has already been related to highlighted antiviral activity [47]. Its infection dependent antiviral mechanisms seem to be related to the number of hydroxyl groups present on the benzene ring and galloyl group. Among the extracts of study, gallocatechin is exclusively present in *Ziziphora hispanica* and *Thymus longiflorus* extracts (in higher abundance in the first plant), which may be associated with their high antiviral activity against FCV. Moreover, rosmarinic acid are also present in both (being in higher abundance in *Thymus* spice). This compound is an ester of caffeic acid, found also in rosemary and spearmint, with two aromatic rings with dihydroxyl groups in its structure. Its structure is possibly related to its demonstrated in vitro and in vivo antiviral activity [48].

Nevertheless, *Origanum bastetanum* seems to be the most consistent antiviral extract, with inhibiting effects on the replication of both FCV and MNV. A relationship between the abundance of phenolic compounds in this sample with a high number of hydroxyl and ester groups in their structures and its synergistic antiviral activity may be assumed. The presence of salvianolic acid, an antiviral agent previously reported against a variety of viruses, to a higher extent in this plant, and which is almost exclusively present in this extract, may also be linked with the presented results [49]. As for other antiviral agents, we can hypothesize on the relation between the inhibition of FCV and MNV of this extract with the presence of compounds with six hydroxyl groups as well as ester groups [4]. Furthermore, catechol groups are also present in a variety of phenolic compounds found in *Origanum bastetanum* extract, such as luteolin, luteolin rutinoside or amburoside A. It has been previously reported that the antiviral activity of these phenolic compounds found in oregano essential oils is related to the damage of the virus capsid for FCV [50]. These findings were also supported by other vegetable samples, such as green tea extract (GTE), on virus like-particles of human norovirus [51]. Even though the relationship between chemical structure and bioactivity of single polyphenols has been considered, the possible synergistic effect between the different phenolic compounds present in each extract may be highly responsible for the observed bioactivity. In fact, the synergistic effect of these great antiviral agents seems to be of great importance in the obtained results for oregano extract.

## 5. Conclusions

The present results demonstrate that MAP species growing in the Granada High Plateau present a high variety of phenolic compounds with great potential as food additives due to their antioxidant and antiviral activities. Extraction conditions were evaluated for obtaining MAP extracts with potential properties. Indeed, plant ethanolic extracts presented high antioxidant activity, with *Origanum bastetanum* being the most promising among the studied herbs, with the best results for all assays, comparable to a rosemary commercial extract. Moreover, *Origanum bastetanum* extract has proven to be successful against norovirus surrogates FCV and MNV, whereas *Ziziphora hispanica* showed the highest effect against FCV. These bioactivities were theorized in relation to their phenolic extracts compounds’ chemical structure. In this sense, the present research reinforces an interest in medicinal and aromatic plants typically found in the Mediterranean region as potential sources of bioactive compounds of important use in the food industry.

## Figures and Tables

**Table 1:** Chemical characterization of aromatic plant extracts.

RT (min)	*m*/*z*	Mass	Score (%)	Error (ppm)	Molecular Formula	Proposed Compound	MAP Extract
**Phenolic acids and derivatives**							
8.834	315.0728	316.0794	98.62	−1.66	C_13_H_16_O_9_	Protocatechuic acid hexoside	TL
9.227	197.0466	198.0528	94.18	−5.11	C_9_H_10_O_5_	Syringic acid	TG, TM, TL, Z
10.259	353.0866	354.0951	94.37	3.7	C_16_H_18_O_9_	Chlorogenic acid isomer	Z
10.571	359.0989	360.1061	83.34	−1.21	C_15_H_20_O_10_	Syringic acid glucoside	OG
11.89	337.0943	338.1009	88.54	−2.02	C_16_H_18_O_8_	Coumaroylquinic acid isomer I	Z
13.922	337.0947	338.1019	90.66	−5.04	C_16_H_18_O_8_	Coumaroylquinic acid isomer II	Z
14.47	367.1046	368.1107	96.75	−2.77	C_17_H_20_O_9_	Feruloylquinic acid	TG, TM, TL
14.493	135.0459	136.0531	84.48	−5.23	C_8_H_8_O_2_	Piceol	Z
19.093	359.0763	360.0845	96.48	2.83	C_18_H_16_O_8_	Rosmarinic acid isomer I	TL, Z
27.498	359.0789	360.0845	92.64	−4.4	C_18_H_16_O_8_	Rosmarinic acid isomer II	TG, TM, TL
31.462	373.0964	374.1002	75.93	−9.08	C_19_H_18_O_8_	Rosmarinic acid methyl esther isomer I	TG, TM
31.625	373.0944	374.1002	93.94	−3.78	C_19_H_18_O_8_	Rosmarinic acid methyl esther isomer II	TG, TM, TL
**Flavonoids**							
12.727	593.1517	594.1588	75.5	−0.59	C_27_H_30_O_15_	Luteolin rutinoside isomer I	All
12.929	305.0722	306.0798	89.04	2.2	C_15_H_14_O_7_	Gallocatechin	TL, Z
12.965	593.1543	594.1585	86.01	−5.24	C_27_H_30_O_15_	Luteolin rutinoside isomer II	All
13.148	305.0721	306.0791	87.21	2.51	C_15_H_14_O_7_	Epigallocatechin	TG, TM
14.207	449.1109	450.1162	92.11	−4.15	C_21_H_22_O_11_	Eriodictyol glucoside	TG, TM, TL
14.818	447.0967	448.1006	79.62	−7.44	C_21_H_20_O_11_	Luteolin glucoside isomer I	TG, TM, TL
15.289	593.1537	594.1585	91.13	−3.87	C_27_H_30_O_15_	Luteolin rutinoside isomer III	TM
16.096	447.097	448.1006	77.34	−8.03	C_21_H_20_O_11_	Luteolin glucoside isomer II	TM, TL
17.063	607.1682	608.1753	72.37	−1.93	C_28_H_32_O_15_	Barosmin	Z
17.831	303.0541	304.0583	77.05	−9.62	C_15_H_12_O_7_	Taxifolin	TG, TM
17.906	445.0785	446.0849	97.64	−1.47	C_21_H_18_O_11_	Apigenin glucuronide	TL
20.305	287.0571	288.0634	96.91	−3.17	C_15_H_12_O_6_	Eriodictyol isomer I	TM, TL
22.323	285.0432	286.0477	79.16	−9.46	C_15_H_10_O_6_	Luteolin	TG, TM, TL, Z
22.428	287.0595	288.0634	72.47	−11.56	C_15_H_12_O_6_	Eriodictyol isomer II	OG, TG
24.173	313.0726	314.0798	83.17	−2.52	C_17_H_14_O_6_	Cirsimaritin isomer I	OG, TG, TM, TL
24.532	329.0676	330.074	96.57	−2.53	C_17_H_14_O_7_	Cirsiliol	TM, TL
25.034	269.0458	270.0528	99.41	−0.76	C_15_H_10_O_5_	Apigenin	TM, TL
25.231	271.0628	272.0685	91.37	−5.61	C_15_H_12_O_5_	Naringenin	TG
25.384	313.074	314.079	86.1	−6.94	C_17_H_14_O_6_	Cirsimaritin isomer II	OG, TM, TL
25.752	329.0685	330.074	91.18	−5.16	C_17_H_14_O_7_	Thymusin	TG, TM
28.401	299.0578	300.0634	91.45	−5.38	C_16_H_12_O_6_	Hispidulin	TM
28.932	313.073	314.079	94.39	−3.35	C_17_H_14_O_6_	Cirsimaritin isomer III	TM, TL
29.414	343.0847	344.0896	85.87	−6.56	C_18_H_16_O_7_	Cirsilineol isomer I	TG, TM, TL
29.97	313.0723	314.079	97.73	−1.45	C_17_H_14_O_6_	Cirsimaritin isomer IV	TM, TL
30.925	343.0839	344.0896	93.48	−4.29	C_18_H_16_O_7_	Cirsilineol isomer II	TM, TL
31.808	283.0625	284.0685	93.61	−4.6	C_16_H_12_O_5_	Genkwanin	TM, TL
**Lignans**							
18.298	555.1127	556.1217	93.67	3.38	C_27_H_24_O_13_	Salvianolic acid K isomer I	TL
20.636	491.0988	492.1056	99.37	−0.5	C_26_H_20_O_10_	Salvianolic acid C	TM, TL
21.242	493.1124	494.1196	93.27	3.41	C_26_H_22_O_10_	Salvianolic acid A isomer I	OG
21.312	717.1441	718.1512	92.68	3.02	C_36_H_30_O_16_	Salvianolic acid B isomer I	OG
21.499	493.1163	494.1213	90.03	−4.42	C_26_H_22_O_10_	Salvianolic acid A isomer II	OG, TL
21.687	493.1144	494.1215	79.06	−0.37	C_26_H_22_O_10_	Salvianolic acid A isomer III	OG
22.891	717.1468	718.1542	97.81	−1.07	C_36_H_30_O_16_	Salvianolic acid B isomer II	OG
**Phenolic glycosides**							
6.728	331.104	332.1112	83.73	−1.28	C_14_H_20_O_9_	Leonuriside A	OG
13.404	583.166	584.173	73.58	1.96	C_26_H_32_O_15_	Seguinoside K	OG
15.91	433.1131	434.1203	79.12	2.39	C_21_H_22_O_10_	Caffeylarbutin	OG
16.153	421.114	422.1209	96.94	0.91	C_20_H_22_O_10_	Amburoside A	OG
**Terpenes**							
17.152	369.1588	370.1628	77.01	−8.89	C_18_H_26_O_8_	Thymohydroquinone acetylglucoside	TG
30.546	455.3567	456.3603	78.84	−7.46	C_30_H_48_O_3_	Ursolic acid/Oleanolic acid	TG
33.476	329.1794	330.1831	73.16	−10.45	C_20_H_26_O_4_	Carnosol	TG, TL
**Others**							
2.903	195.0478	196.055	50.7	−13.2	C_13_H_8_O_2_	Xanthone	TL, Z
3.056	191.0213	192.027	75.04	−8.23	C_6_H_8_O_7_	Isocitric acid	TL, Z
3.103	149.0085	150.0157	82.53	5.1	C_4_H_6_O_6_	Tartaric acid	Z
3.166	179.0571	180.0634	94.8	−5.37	C_6_H_12_O_6_	Glucose	TL
3.436	133.0135	134.021	45.58	4.12	C_4_H_6_O_5_	Malic acid	All
4.623	191.0203	192.027	97.97	−3.01	C_6_H_8_O_7_	Citric acid	TL, Z
5.414	147.0304	148.0377	85.56	−3.41	C_5_H_8_O_5_	Pentonic acid lactone	Z
9.604	447.1532	448.1581	88.89	−5.12	C_19_H_28_O_12_	Barlerin	TM, TL
12.043	329.1243	330.1315	47.6	−0.2	C_15_H_22_O_8_	Dihydrocaffeyl alcohol glucopyranoside	OG
12.48	367.1047	368.1119	80.21	−3.2	C_17_H_20_O_9_	Caffeoylquinic acid methyl ester	Z
12.774	387.1684	388.1756	71.31	−5.73	C_18_H_28_O_9_	Tuberonic acid glucoside isomer I	TG, TL
13.013	387.1676	388.1748	94.26	−3.8	C_18_H_28_O_9_	Tuberonic acid glucoside isomer II	OG, TM, TL, Z
13.973	179.0354	180.0423	98.21	−1.83	C_9_H_8_O_4_	Caffeic acid	TG, TM, TL
14.074	659.1614	660.1687	98.32	0.52	C_31_H_32_O_16_	Dicaffeoyl-hydroxy-methylglutaroyl-quinic acid	OG
14.513	401.1832	402.1904	77.65	−3.53	C_19_H_30_O_9_	Tuberonic acid methyl esther glucoside	Z
15.019	225.1151	226.1223	74.35	−7.74	C_12_H_18_O_4_	Tuberonic acid	TG, TM
19.981	401.2207	402.2278	67.62	−6.13	C_20_H_34_O_8_	Botcinic acid	Z
23.503	327.2198	328.227	73.43	−6.11	C_18_H_32_O_5_	Polyrhacitide A	Z
23.713	327.2185	328.2258	83.25	−2.4	C_18_H_32_O_5_	Trihydroxyoctadecadienoic acid	OG, TG, TM, TL
24.955	329.235	330.2406	92.2	−4.88	C_18_H_34_O_5_	Pinellic acid isomer I	TG, TM, TL
25.252	329.2359	330.2431	68.98	−7.42	C_18_H_34_O_5_	Pinellic acid isomer II	TG
30.446	165.0933	166.0994	92.18	−7.15	C_10_H_14_O_2_	Cymenediol	TG

**Table 2:** In vitro antioxidant activity of extracts of aromatic plants obtained with different hydroethanolic mixtures determined by FRAP, TEAC and ORAC assays.

Antioxidant Assay	Solvent (% EtOH)	*Origanum bastetanum*	*Thymus zygis gracilis*	*Thymus membranaceus*	*Thymus longiflorus*	*Ziziphora hispánica*
TEAC (µmol Trolox eq./mg dry extract)	100	3 ± 1	4 ± 3	3.1 ± 0.7	2.3 ± 0.5	1.2 ± 0.4
	80	8.5 ± 0.7	7.3 ± 1.5	4 ± 1	7.1 ± 1.0	1.7 ± 0.6
	50	3 ± 1	4 ± 2	1.1 ± 0.2	2.5 ± 0.3	0.66 ± 0.07
	0	0.5 ± 0.1	0.4 ± 0.2	0.9 ± 0.3	0.4 ± 0.1	0.3 ± 0.1
FRAP (µmol FeSO_4_ eq./mg extract)	100	0.64 ± 0.07	1.4 ± 0.1	0.75 ± 0.05	0.46 ± 0.07	0.33 ± 0.07
	80	2.2 ± 0.1	1.5 ± 0.1	1.4 ± 0.1	1.7 ± 0.1	0.38 ± 0.05
	50	1.6 ± 0.1	1.23 ± 0.07	1.2 ± 0.1	2.1 ± 0.1	0.49 ± 0.04
	0	0.42 ± 0.09	0.39 ± 0.09	0.32 ± 0.08	0.95 ± 0.06	0.15 ± 0.010
ORAC (µmol Trolox eq./mg dry extract)	100	2.20	4.33	3.21	2.02	1.69
	80	5.31	3.81	3.72	3.56	2.07
	50	3.56	4.13	3.35	3.79	3.37
	0	1.62	1.06	0.84	1.47	1.38

## Data Availability

Data is contained within the article or supplementary material.

## Supplementary Materials

The following supporting information can be downloaded at: https://www.mdpi.com/article/10.3390/foods11131862/s1. Table S1: Altitude, maximum, minimum and medium temperature averages, water rain and solar radiation of the recollection months registered in the Granada´s High Plateau area. Table S2: Extraction yields (%) of conventional extraction of aromatic plants using different extraction solvent composition. The % extraction yield is the g of extract obtained per g of plant expressed in percentage. Table S3: Area of compounds tentatively identified for each of the studied extracts, expressed as mean value ± standard deviation.
