# Supplementary material for: Potential Antioxidant and Antiviral Activities of Hydroethanolic Extracts of Selected Lamiaceae Species
Source: Foods. 2022 Jun 24;11(13):1862. doi: 10.3390/foods11131862 (PMC9265503; doi:10.3390/foods11131862)
Supplement: Supplementary file 1 [file foods-11-01862-s001.zip › foods-1763301-supplementary.pdf]

Table S1. Altitude, maximum, minimum and medium temperature averages, water rain and solar radiation of the recollection months registered in the Granada´s High Plateau area.

**Parameter**	Recollection month		Drying period
	**April**	**May**	**June**
Altitude (m)		500 - 2382	
Maximum temperature avergage (ºC)	21.1	27.9	33.9
Minimum temperature avergage (ºC)	2.5	9.2	14.9
Mediuum temperature avergage (ºC)	13.3	18.5	24.4
Water rain (mm)	17.8	0.4	0
Global radiation on horizontal surface (MJ/m²)	642.3	743.2	810.4
Global radiation on horizontal surface (MWh/m²)	178.3	206.3	225.3

Table S2. Extraction yields (%) of conventional extraction of aromatic plants using different extraction solvent composition. The % extraction yield is the g of extract obtained per g of plant expressed in percentage.

MAP	Solvent			
	**EtOH 100%**	**EtOH-H_20 (80:20, v/v)**	**EtOH-H_20 (50:50, v/v)**	**H_20 100%**
Origanum bastetanum	8.4	44.5	65.6	64.1
Thymus zygis gracilis	6.9	18.3	19.4	20.9
Thymus membranaceus	10.9	35.2	48.1	38.0
Thymus longiflorus	25.5	30.5	26.7	31.6
Ziziphora hispanica	13.8	26.8	38.9	51.4

Table S3. Area of compounds tentatively identified for each of the studied extracts, expressed as mean value ± standard deviation.

Proposed Compound	OG	TG	TL	TM	Z
**Phenolic acids and derivatives**					
**Protocatechuic acid hexoside**	ND	ND	$4,3x10^6$ ± $2x10^5$	ND	ND
**Syringic acid**	ND	$1,3x10^7$ ± $2x10^6$	$4x10^6$ ± $1x10^6$	$4,4x10^6$ ± $6x10^5$	$4,3x10^6$ ± $8x10^5$
**Chlorogenic acid isomer**	ND	ND	ND	ND	$2,4x10^7$ ± $2x10^6$
**Syringic acid glucoside**	$8x10^6$ ± $6x10^6$	ND	ND	ND	ND
**Coumaroylquinic acid isomer I**	ND	ND	ND	ND	$3,1x10^7$ ± $5x10^6$
**Coumaroylquinic acid isomer II**	ND	ND	ND	ND	$1,42x10^7$ ± $4x10^5$
**Feruloylquinic acid**	ND	$8x10^6$ ± $1x10^6$	$9,8x10^6$ ± $2x10^5$	$5x10^6$ ± $1x10^6$	ND
**Piceol**	ND	ND	ND	ND	$1,5x10^7$ ± $4x10^6$
**Rosmarinic acid isomer I**	ND	ND	$2,4x10^7$ ± $1x10^6$	ND	$1,4x10^7$ ± $3x10^6$
**Rosmarinic acid isomer II**	ND	$6x10^6$ ± $5x10^6$	$8x10^5$ ± $2x10^5$	$1,6x10^7$ ± $3x10^6$	ND
**Rosmarinic acid methyl esther isomer I**	ND	$9x10^6$ ± $2x10^6$	ND	$8x10^6$ ± $5x10^6$	ND
**Rosmarinic acid methyl esther isomer II**	ND	$5x10^5$ ± $4x10^5$	$2,7x10^6$ ± $3x10^5$	$1,6x10^7$ ± $1x10^6$	ND
**Flavonoids**					
**Luteolin rutinoside isomer I**	$1,6x10^6$ ± $1x10^5$	$4x10^6$ ± $1x10^6$	$5x10^6$ ± $2x10^6$	$7x10^6$ ± $1x10^6$	$6x10^6$ ± $2x10^6$
**Gallocatechin**	ND	ND	$1,4x10^7$ ± $8x10^6$	ND	$2x10^7$ ± $1x10^7$

**Luteolin rutinoside isomer II**	5x10^{5} ± 1x10^{5}	4x10^{6} ± 1x10^{6}	1,3x10^{5} ± 1x10^{4}	2,2x10^{6} ± 2x10^{5}	1,1x10^{5} ± 4x10^{4}
**Epigallocatechin**	ND	1x10^{7} ± 1x10^{6}	ND	4,3x10^{7} ± 8x10^{6}	ND
**Eriodictyol glucoside**	ND	8x10^{6} ± 2x10^{6}	4,58x10^{6} ± 1x10^{4}	3,2x10^{6} ± 4x10^{5}	ND
**Luteolin glucoside isomer I**	ND	7x10^{6,4} ± 2x10^{5}	4,5x10^{6} ± 4x10^{5}	5x10^{6} ± 2x10^{5}	ND
**Luteolin rutinoside isomer III**	ND	ND	ND	2,2x10^{6} ± 3x10^{5}	ND
**Luteolin glucoside isomer II**	ND	ND	3,4x10^{6} ± 4x10^{5}	2,9x10^{6} ± 2x10^{5}	ND
**Barosmin**	ND	ND	ND	ND	
**Taxifolin**	ND	7,2x10^{6} ± 1x10^{5}	ND	7x10^{6} ± 3x10^{5}	ND
**Apigenin glucuronide**	ND	ND	2,2x10^{6} ± 3x10^{5}	ND	ND
**Eriodictyol isomer I**	ND	ND	6x10^{6} ± 1x10^{6}	1,27x10^{7} ± 4x10^{5}	ND
**Luteolin**	ND	1,74x10^{7} ± 1x10^{6}	6x10^{6} ± 1x10^{6}	1,4x10^{7} ± 1x10^{6}	3x10^{6} ± 1x10^{6}
**Eriodictyol isomer II**	2,8x10^{6} ± 3x10^{5}	1,4x10^{7} ± 1x10^{6}	ND	ND	ND
**Cirsimaritin isomer I**	5,4x10^{6} ± 4x10^{5}	5,4x10^{6} ± 1x10^{5}	6,1x10^{6} ± 4x10^{5}	1,7x10^{7} ± 1x10^{6}	ND
**Cirsiliol**	ND	ND	1,6x10^{6} ± 4x10^{5}	6x10^{6} ± 1x10^{6}	ND
**Apigenin**	ND	ND	2,2x10^{6} ± 5x10^{5}	9x10^{6} ± 1x10^{6}	ND
**Naringenin**	ND	8x10^{6} ± 1x10^{6}	ND	ND	ND
**Cirsimaritin isomer II**	5,8x10^{6} ± 5x10^{5}	ND	7x10^{6} ± 1x10^{6}	1,6x10^{7} ± 1x10^{6}	ND
**Thymusin**	ND	3x10^{6} ± 3x10^{6}	ND	7x10^{6} ± 1x10^{6}	ND
**Hispidulin**	ND	ND	ND	5x10^{6} ± 1x10^{6}	ND
**Cirsimaritin isomer III**	ND	ND	1,6x10^{6} ± 2x10^{5}	2,6x10^{7} ± 3x10^{6}	ND
**Cirsilineol isomer I**	ND	2x10^{6} ± 2x10^{6}	7x10^{5} ± 2x10^{5}	7x10^{6} ± 1x10^{6}	ND
**Cirsimaritin isomer IV**	ND	ND	7x10^{5} ± 1x10^{5}	9x10^{6} ± 1x10^{6}	ND
**Cirsilineol isomer II**	ND	ND	1,6x10^{7} ± 2x10^{6}	7,7x10^{7} ± 5x10^{6}	ND

**Genkwanin**	ND	ND	$2,24x10^6$ ± $5x10^4$	$2,1x10^7$ ± $3x10^7$	ND
			**Lignans**		
**Salvianolic acid K isomer I**	ND	ND	$3x10^6$ ± $2x10^6$	ND	ND
**Salvianolic acid C**	ND	ND	$7,6x10^6$ ± $3x10^5$	$9x10^6$ ± $1x10^6$	ND
**Salvianolic acid A isomer I**	$1x10^7$ ± $2x10^6$	ND	ND	ND	ND
**Salvianolic acid B isomer I**	$2,7x10^6$ ± $6x10^5$	ND	ND	ND	ND
**Salvianolic acid A isomer II**	$8x10^6$ ± $5x10^6$	ND	$4x10^6$ ± $1x10^6$	ND	ND
**Salvianolic acid A isomer III**	$5x10^6$ ± $2x10^6$	ND	ND	ND	ND
**Salvianolic acid B isomer II**	$6,4x10^6$ ± $5x10^5$	ND	ND	ND	ND
			**Phenolic glycosides**		
**Leonuriside A**	$5,2x10^6$ ± $5x10^5$	ND	ND	ND	ND
**Seguinoside K**	$9,6x10^5$ ± $5x10^4$	ND	ND	ND	ND
**Caffeylarbutin**	$3,8x10^6$ ± $1x10^5$	ND	ND	ND	ND
**Amburoside A**	$3,3x10^7$ ± $1x10^5$	ND	ND	ND	ND
			**Terpenes**		
**Thymohydroquinone acetylglucoside**	ND	$7x10^6$ ± $5x10^5$	ND	ND	ND
**Ursolic acid/Oleanolic acid**	ND	$9x10^5$ ± $8x10^5$	ND	ND	ND
**Carnosol**	ND	$2x10^7$ ± $2x10^7$	$1,7x10^6$ ± $1x10^5$	ND	ND
			**Others**		
**Xanthone**	ND	ND	$3x10^6$ ± $1x10^6$	ND	$1,10x10^7$ ± $3x10^6$

**Isocitric acid**	ND	ND	$6x10^6 \pm 5x10^6$	ND	$1,25x10^7 \pm 3x10^5$
**Tartaric acid**	ND	ND	ND	ND	$1,90x10^7 \pm 3x10^6$
**Glucose**	ND	ND	$3,8x10^6 \pm 1x10^5$	ND	ND
**Malic acid**	$3,7x10^6 \pm 5x10^5$	$5x10^6 \pm 1x10^6$	$1,1x10^7 \pm 1x10^6$	$4,1x10^6 \pm 4x10^5$	$1,00x10^7 \pm 2x10^6$
**Citric acid**	ND	ND	$5x10^6 \pm 4x10^6$	ND	$7,00x10^6 \pm 6x10^6$
**Pentonic acid lactone**	ND	ND	ND	ND	$1,3x10^7 \pm 2x10^6$
**Barlerin**	ND	ND	$2,9x10^6 \pm 3x10^5$	$8,5x10^6 \pm 3x10^5$	ND
**Dihydrocaffeyl alcohol glucopyranoside**	$1x10^6 \pm 2x10^5$	ND	ND	ND	ND
**Caffeoylquinic acid methyl ester**	ND	ND	ND	ND	$1,06x10^7 \pm 4x10^4$
**Tuberonic acid glucoside isomer I**	ND	$1,2x10^7 \pm 1x10^7$	$2x10^7 \pm 1x10^7$	ND	ND
**Tuberonic acid glucoside isomer II**	$1,5x10^7 \pm 1x10^6$	ND	$3,2x10^7 \pm 1x10^6$	$2,7x10^6 \pm 1x10^5$	$1,3x10^7 \pm 1x10^6$
**Caffeic acid**	ND	$6x10^{6,5} \pm 5x10^5$	$1,7x10^6 \pm 3x10^5$	$4,8x10^6 \pm 4x10^5$	ND
**Dicaffeoyl-hydroxy-methylglutaroyl-quinic acid**	$1,1x10^7 \pm 5x10^6$	ND	ND	ND	ND
**Tuberonic acid methyl esther glucoside**	ND	ND	ND	ND	$1,3x10^7 \pm 9x10^6$
**Tuberonic acid**	ND	$4x10^{6,3} \pm 1x10^5$	ND	$4x10^6 \pm 3x10^5$	ND
**Botcinic acid**	ND	ND	ND	ND	$1,2x10^7 \pm 4x10^6$
**Polyrhacitide A**	ND	ND	ND	ND	$8x10^6 \pm 4x10^6$

**Trihydroxyoctadecadienoic acid**	$6,74x10^6 \pm 4x10^4$	$8,2x10^6 \pm 9x10^5$	$5,5x10^6 \pm 3x10^5$	$1,32x10^7 \pm 3x10^5$	ND
**Pinellic acid isomer I**	ND	$8,4x10^6 \pm 5x10^5$	$4,2x10^6 \pm 2x10^5$	$8x10^6 \pm 1x10^6$	ND
**Pinellic acid isomer II**	ND	$4x10^6 \pm 3x10^6$	ND	ND	ND
**Cymenediol**	ND	$2x10^7 \pm 2x10^7$	ND	ND	ND
